# Sequential, but not Concurrent, Incubation of Cathepsin K and L with Type I Collagen Results in Extended Proteolysis

**DOI:** 10.1038/s41598-019-41782-1

**Published:** 2019-04-01

**Authors:** Akia N. Parks, Juhi Nahata, Naomi-Eliana Edouard, Johnna S. Temenoff, Manu O. Platt

**Affiliations:** 1 W.H. Coulter Department of Biomedical Engineering, Georgia Institute of Technology and Emory University, 313 Ferst Dr NW, Atlanta, GA 30332 USA; 2 Mathematics Department, Spelman College, 350 Spelman Ln, Atlanta, GA 30314 USA; 3 Petit Institute for Bioengineering and Bioscience, Georgia Institute of Technology, 315 Ferst Dr NW, Atlanta, GA 30332 USA

## Abstract

Degradation of extracellular matrix (ECM) during tendinopathy is, in part, mediated by the collagenolytic cathepsin K (catK) and cathepsin L (catL), with a temporal component to their activity. The objective of this study was to determine how catK and catL act in concert or in conflict to degrade collagen and tendon ECM during tissue degeneration. To do so, type I collagen gels or ECM extracted from apolipoprotein E deficient mouse Achilles tendons were incubated with catK and catL either concurrently or sequentially, incubating catK first, then catL after a delayed time period. Sequential incubation of catK then catL caused greater degradation of substrates over concurrent incubation, and of either cathepsin alone. Zymography showed there were reduced amounts of active enzymes when co-incubated, indicating that cannibalism, or protease-on-protease degradation between catK and catL was occurring, but incubation with ECM could distract from these interactions. CatK alone was sufficient to quickly degrade tendon ECM, but catL was not, requiring the presence of catK for degradation. Together, these data identify cooperative and conflicting actions of cathepsin mediated collagen matrix degradation by considering interactive effects of multiple proteases during tissue degeneration.

## Introduction

Tendinopathy, also known as tendon overuse, is the most common tendon injury and can cause significant pain and limited range of motion. One of the clinical hallmarks of tendinopathy is the degeneration of tendon extracellular matrix (ECM)^1–3^. If left untreated, early tendon injury can progress to full tendon rupture, concomitant degeneration of other joint tissues, and abnormal joint mechanics^1,4–8^. Multiple etiological factors of tendinopathy contribute to pathological tendon degeneration, including mechanical overload that can cause microtrauma, and dysregulated expression of proteases and their endogenous inhibitors by resident tenocytes^1,8–10^. Type I collagen accounts for 95% of the total collagen content in tendon with small amounts of types III and V^1,8,11–13^. Other tendon components include approximately 2% elastin and 1–2% proteoglycans, largely decorin and byglycan^1,6,8,12,14^.

Collagenolytic proteases are important to tendon matrix homeostasis by facilitating normal collagen turnover and ECM remodeling during wound healing^8,15,16^. However, upregulated collagenases, namely cysteine cathepsins and matrix metalloproteinases (MMP), have been reported in tendinopathic progression^8,9,15–21^. Cysteine cathepsins are a family of proteases, implicated in numerous tissue destructive diseases, including orthopaedic tissues^22–27^. CatK is the most potent mammalian collagenase, able to cleave the collagen molecule at multiple sites within the triple helix and at the telopeptide ends^23^. CatK is primarily responsible for normal osteoclastic bone resorption in both humans and mice^23,28^, however, elevated catK mRNA levels were also measured in human calcified tendon tissues^29^. Increased catK expression was also found in a rabbit model of flexor tendon injury^30^. Human cathepsin V (catV) is the most potent mammalian elastase with some collagenolytic activity, and is orthologous to murine cathepsin L (catL). CatL is ubiquitously expressed in mice and was found to be upregulated in diabetes and cardiovascular diseases^31–34^.

In previous studies, we demonstrated upregulated active cathepsins in a treadmill-based rat model of supraspinatus overuse injury^7,21^. There was an upregulation of catK and catL in the tendons after 4 weeks of overuse, and catL remained upregulated after 8 weeks of overuse, with evidence of tissue damage to tendon and articular cartilage by 10 weeks^7,21^. This production of cathepsins at different stages of the tendon injury led to the question of whether the temporal differences in catK and catL activity correlated with the tissue damage, and whether there was cooperation or synergistic effects specific to the timing of the presence of these active proteases.

Previous studies have investigated how multiple proteases, within and between families, work synergistically to degrade a shared substrate. One study showed that cathepsins L, B, S, and F were able to fully degrade type I collagen fragments (in the presence of chondroitin-4 sulfate) only after first being cleaved by MMP-1^32^. Increased degradation of type I collagen fibers with age-related modifications by catK have also been reported after the fibers were initially cleaved by MMP-1, MMP-8, and MMP-13^35^. Additionally, concurrent and sequential combinations of cathepsins B, L, D and calpain demonstrated varied synergistic capacities to degrade and disassemble myofibrillar protein of grass carp, suggesting that multiple cysteine proteases in sequence may be driving increased rate of tissue degradation^36^. CatK is subject to autodigestion, or self-cleavage over time^37^, and we have demonstrated that cathepsins can hydrolyze other cathepsins, even in the presence of a substrate, a phenomenon we termed cathepsin cannibalism^37,38^. These studies suggest that cooperative substrate cleavage, autodigestion, and cannibalism among multiple proteolytic enzymes can dictate the amount of active proteases in a system, impacting rate and amount of tissue degradation. However, it is still unclear how multiple cathepsins, present simultaneously, impact tendon ECM degradation, particularly type I collagen.

In this study we employed polymerized collagen gel and homogenized murine tendon ECM models as test beds to investigate how catK and catL act in concert to degrade these collagen matrix substrates. These experimental systems were used to test the hypothesis that catK initiation of substrate degradation primes the substrates by exposing sites susceptible to further hydrolysis by catL, and thus, pre-incubating matrix substrate with catK before introducing catL, resulting in greater hydrolysis than when concurrently co-incubated with catK and catL.

## Methods

### Mouse Achilles Tendon Isolation and ECM Preparation

All animal procedures were approved by the Georgia Institute of Technology Institutional Animal Care and Use Committee. All methods were performed in accordance with the relevant guidelines and regulations. C57/Bl6 mice deficient in apolipoprotein E were obtained from Jackson Laboratory (Bar Harbor, ME). These animals were chosen because they share the background genotype with cathepsin K deficient animal models planned for use in future studies and for other organ system studies, but the double transgenic colony was lost and is being regenerated. Mice were bred and progeny were raised to 2–4 months of age and then sacrificed. The left and right Achilles tendons were extracted from 37 mice. Each tendon was separated from the gastrocnemius muscle tissue and bluntly cut at the calcaneous insertion point. Tendons were then frozen in PBS at −20 °C until needed for homogenization.

To prepare tendon ECM, Achilles tendons were dissected from mice and homogenized in zymography lysis buffer with 0.1 mM leupeptin in tissue grinding microcentrifuge tubes with resin (Sigma-Aldrich, St. Louis, MO), and then centrifuged to remove the Triton-X soluble fraction and retaining the insoluble tendon ECM extract. These remaining insoluble, homogenized tendon ECM were pooled from a number of mice, well mixed, washed in phosphate buffer, pH 6, and redistributed into separate microcentrifuge tubes for cathepsin incubation, including no-cathepsin controls. The buffer was aspirated prior to incubations with enzyme.

### Collagen Gels Preparation

Soluble rat tail type I collagen which may contain glycosaminoglycans as isolated from native tissue (Gibco/Thermo, Waltham, MA) at 3 mg/mL was combined with deionized water, 10X phosphate-buffered sulfate (PBS), and 1 N NaOH to obtain a 2 mg/mL solution at pH between 6.5 and 7.5. 50 µL collagen gels were polymerized in 1.5 mL microcentrifuge tubes. After polymerization, gels were washed in a 0.1 M sodium phosphate buffer with 2 mM dithiothreitol (DTT) and 1 mM ethylenediaminetetraacetic acid (EDTA) at pH6, hereafter referred to as pH6 assay buffer.

### Concurrent and sequential cathepsin incubations

Recombinant human cathepsin K (catK) (ENZO, Farmingdale, NY) and recombinant mouse cathepsin L (catL) (Novoprotein, Summit, NJ,) were used. Human catK was used because there is no commercially available murine catK, and their catalytic activity is similar although the catK between these two species display pointed differences in response to certain inhibitors^39^. CatK and catL were incubated at a 0.2 µM concentration in pH6 assay buffer with collagen gels or tendon ECM, prepared as described above, in addition to a no-cathepsin control. All incubations were conducted at 37 °C with slight agitation for 8 hrs total. CatK and catL were incubated individually with collagen gels or tendon ECM, concurrently, with the same start point, or in sequential combinations, where the primary cathepsin was pre-incubated alone for 4 hrs and then the secondary cathepsin was added and incubated for an additional 4 hrs, for a total of 8 hrs time period. After the incubation period, leupeptin was added to each sample as a weak binding cathepsin inhibitor to a final concentration of 0.1 mM to stop the reaction. All samples were centrifuged at 19,090 × g for 15 min at 25 °C. The supernatant was collected and referred to as the soluble fraction; equal volumes of the soluble fraction were loaded for electrophoresis. The pellet fraction was also collected and reconstituted with equal volumes of zymography lysis buffer (50 µl) containing 0.1 mM leupeptin. Relative amounts of soluble and insoluble fractions loaded are the same for the tendon ECM conditions. For collagen gel conditions there was a slight difference since the collagen gels are hydrogels and yield a slightly higher supernatant volume because of this; equal volumes from supernatant were loaded for each gel. Insoluble fractions from collagen gels were reconstituted in same volumes of zymography lysis buffer as tendon ECM experiments. Equal volumes from those aliquots loaded for analysis by SDS-PAGE, zymography, and Western blots.

### SDS-PAGE and Western Blotting

In preparation for electrophoresis, each soluble fraction and remaining pellet fraction were prepared with 5X reducing loading dye for SDS-polyacrylamide gel electrophoresis (SDS-PAGE) and Western blotting. For Western blot, equal volumes of each sample were loaded into 12.5% polyacrylamide gels and separated by molecular weight. After separation, samples from each gel were transferred onto a nitrocellulose membrane. The gels were stained with Coomassie Blue and destained, revealing bands indicative of type I collagen α1 and α2 chains, and β-chains, indicating an α-chain dimer^32,40–44^. Membranes were blocked with Odyssey blocking buffer, (LI-COR Biosciences, Lincoln, NE) then incubated with primary rabbit anti-human catK polyclonal antibody (Protein Tech, Rosemont, IL) or goat anti-mouse catL monoclonal antibody (R&D Systems, Minneapolis, MN) followed by fluorophore conjugated secondary antibody (LI-COR Biosciences, Lincoln, NE) and imaged.

### Multiplex cathepsin zymography

In preparation for electrophoresis, each soluble fraction and remaining pellet fraction were prepared with a 5X non-reducing loading buffer for zymography. Zymography was performed as previously described^45,46^. Briefly, equal volumes of each sample were loaded into 12.5% SDS-PAGE gels embedded with a 0.2% gelatin substrate. After electrophoresis, gels were incubated in renaturing buffer to allow the cathepsins to refold into their native confirmations. Then gels were incubated in sodium acetate buffer, pH 4, or sodium phosphate buffer, pH 6, with 2 mM DTT and 1 mM EDTA for 18 hrs at 37 °C to allow the cathepsins to degrade the embedded gelatin substrate. After incubation, the gels were stained with Coomassie Blue and destained to reveal cleared white bands where active cathepsins degraded the substrate. Gels were imaged with the ImageQuant LAS 4000 (GE Healthcare, Little Chalfont, United Kingdom). Densitometry analysis was performed on the obtained images using ImageJ (NIH).

### Statistical analysis

Statistical significance for SDS-PAGE, zymography, and Western blot data were determined by a one-way ANOVA of normally distributed data in GraphPad Prism between each normalized incubation group with each replicate repeated three times for each condition and statistical significance considered when p < 0.05. All error bars represent the standard error of the mean (SEM).

## Results

### Sequential co-incubation of catK then catL cleaved more type I collagen and tendon ECM than concurrent co-incubation

To test the hypothesis that priming matrix substrates with catK before the addition of catL would yield a greater amount of collagen cleaved compared to matrix co-incubated with catK and catL, collagen gels were incubated with either catK or catL alone, incubated with catK and catL added concurrently, or incubated with sequential catK then catL where the matrix substrates were primed with catK for 4 hrs prior to adding catL for the remaining 4 hrs of the 8 hr incubation period. After the incubation period, each sample was centrifuged and separated into the soluble (matrix fragment and free cathepsins) and pellet (insoluble matrix and bound cathepsins) fractions. Collagen cleavage in the pellet fractions and degradation in the soluble fractions was assessed with Coomassie stained SDS-PAGE. The amount of collagen cleaved is expressed as percent of collagen fragments present in the Coomassie stained polyacrylamide gel quantified with densitometry, normalized to control conditions of collagen gel or tendon ECM incubated in the absence of any proteases over the 8 hr period.

**Figure 1:**
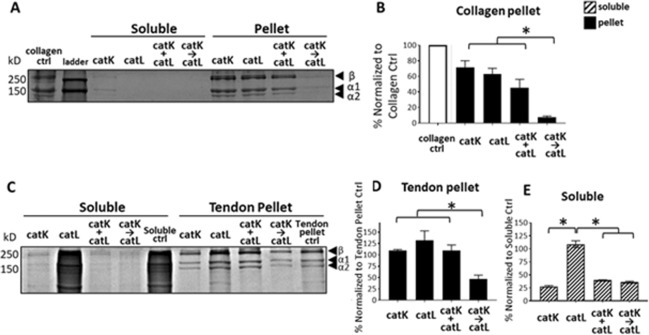
Matrix substrate co-incubated with sequential catK then catL yielded greater collagen cleavage than concurrently incubated catK and catL. Collagen gels and tendon ECM were incubated with catK alone, catL alone, and catK co-incubated with catL concurrently (+), or sequentially (→) for 8 hrs. After centrifugation, SDS-PAGE was conducted on the soluble and pellet fractions of each experiment. SDS-PAGE showed the alpha chains from the collagen pellet with little to no collagen remaining in the soluble fraction after 8 hr cathepsin incubations. (**A**) Collagen control sample was just the collagen gel incubated for same length of time, but in the absence of any added cathepsins. This is the pelleted gel as there was little to no collagen in soluble fraction in the absence of enzyme. Sequentially incubated catK and catL (→) cleaved significantly more collagen than catK concurrently co-incubated with catL (+), or either enzyme alone. (**B**) SDS-PAGE run on the tendon ECM showed fragments released into the soluble fraction and the remaining alpha chains in the pellet after incubation. (**C**) Tendon pellet control sample was just tendon ECM, incubated in buffer for same length of time, but in the absence of any added cathepsins. Soluble fraction collected from this control was prepared as other soluble fractions and labeled the soluble ctrl. In the tendon ECM pellet fraction, similarly to the collagen pellet, SDSPAGE showed significantly greater cleavage with sequential co-incubation (→) than catK alone, catL alone, or concurrent co-incubation of catK and catL (+). (**D**) In the soluble fraction, catL alone was not able to degrade the ECM fragments, leaving significantly more fragments than catK alone, concurrent (+) and sequential (→) catK and catL. (**E**) n = 3, *indicates statistical significance, p < 0.05 as determined by a one-way ANOVA. Error bars represent SEM.

After incubation with the collagen gel, little to no collagen fragments were detected in the soluble fraction for any of the four conditions, including the no-enzyme collagen gel control. In the remaining collagen pellet fraction, α1(I), α2(I), and β-chains (α-chain dimers) were visible in all four conditions (Fig. 1A). After normalizing to the no-cathepsin control that is equivalent to the pellet fractions of the enzyme incubated conditions, 93% of the collagen chains were cleaved in the sequential condition, significantly more than when catK or catL were incubated with collagen gels alone (29% and 37% cleaved, respectively) or co-incubated with collagen gels concurrently (55% cleaved) (Fig. 1B, n = 3, p < 0.05).

Type I collagen gels were used initially as collagen is the major component in tendons, but to determine if sequential cleavage by catK then catL had effects on cleavage of tendon ECM, containing additional ECM protein constituents, we repeated these studies on homogenized mouse Achilles, or just with control groups of soluble tendon ECM incubated only in assay buffer, pH 6, and no cathepsins. Many soluble tendon ECM fragments were present at multiple molecular sizes (Fig. 1C, soluble control), due to the partial solubility of collagen in the pH6 assay buffer^46,47^. After centrifugation to separate control tendon pellet ECM from soluble fragments, the tendon pellet control showed similar α- and β-chains as seen with the collagen gels after SDS-PAGE. Priming with catK followed by incubation with catL, resulted in significantly higher cleavage of matrix from the pellet fraction, with 53% loss of collagen alpha chains (Fig. 1D,E). For the soluble fraction, incubation with catK degraded significantly more collagen than in the absence of catK, regardless of priming, concurrent, or catK alone. When catL was incubated alone with tendon ECM, there was no degradation of the soluble ECM fragments (Fig. 1C,E).

### Cathepsins remained present and active, and display cannibalistic behaviors after 8 hr incubations with collagen gels

**Figure 2:**
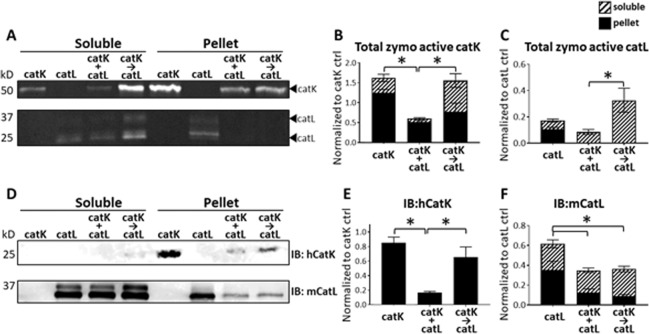
Sequential co-incubation of catK then catL with collagen gels sustained active catK, while bi-directional cannibalism of catK and catL occurred during concurrent co-incubation. Zymography and Western blots for catK and catL were conducted to measure the amount of active catK and catL remaining after incubation with collagen gels. CatK and catL were active (**A**) after 8 hr incubation with collagen as indicated by zymography in the soluble and pellet fractions. Significantly more catK was active when collagen was sequentially co-incubated with catK then catL (→) than concurrent co-incubation (+). (**B**) More catL was active when collagen was primed with catK (→) than concurrent co-incubation with catK (+). (**C**) CatK Western blot detected no catK in the soluble fraction, but in the pellet fraction less total catK remained after concurrent co-incubation with catL (+), suggesting catL was hydrolyzing catK (**D**,**E**). By Western, less total catL was present when co-incubated with catK, even when sequentially co-incubated with catK (→), suggesting catK may also have been hydrolyzing catL. (**D**,**F**) n = 3, *indicates statistical significance, p < 0.05 as determined by a one-way ANOVA. Error bars represent SEM.

After observing that catK priming collagen I, followed by catL cleavage, yielded significantly higher degradation of type I collagen and tendon ECM from the pellet fraction than when concurrently co-incubated, we next investigated functional dynamics between catK and catL responsible for this difference. Active cathepsins are subject to autodigestion, or self-cleavage over time, cathepsin-on-cathepsin hydrolysis, a condition we termed cathepsin cannibalism, and other mechanisms that could be inactivating without protein cleavage, such as oxidation and pH-mediated denaturation^37^; any of these processes could reduce the amount of active cathepsins present in the system, reducing total substrate degradation. We tested the hypothesis that catK or catL were cannibalizing one another in the concurrent co-incubation condition, reducing matrix degradation compared to when catK was pre-incubated with the matrix prior to addition of catL, first using collagen gels. To measure the amount of active cathepsins remaining after the matrix substrate incubation period, zymography was performed on the soluble and reconstituted collagen pellet fractions. Zymography showed active catK remained after the 8 hr incubation period in both the pellet and soluble fractions after collagen gel incubation (Fig. 2A), however, there was significantly higher amounts of active catK remaining when it was incubated, in the absence of catL, added neither concurrently nor sequentially (n = 3, p < 0.05 by one-way ANOVA), as quantified in Fig. 2B,C. These data suggest that in the presence of collagen, catK was stabilized, and protected from autodigestion during this 8 hr period; this was confirmed by Western blot for catK protein (Fig. 2D). There was a significant loss of catK protein when incubated with catL, either concurrently or sequentially, suggesting cannibalistic interactions of catL on catK. This loss of catK was significantly reduced in the sequential case compared to the concurrent co-incubation condition (Fig. 2D,E; n = 3, p < 0.05), indicating greater amount of catK remained and was active under the sequential conditions than concurrent incubation system.

Active catL, detected by zymography, also showed a loss in the amount of active catL in the collagen pellet fraction when co-incubated with catK either concurrently or sequentially (Fig. 2A,C). This was confirmed by significantly more immunodetectable catL protein when incubated alone than when either concurrently or sequentially co-incubated with catK and the collagen gels (Fig. 2F). Together these results suggest bidirectional cathepsin cannibalism, with catK hydrolyzing catL and catL hydrolyzing catK. CatK also preferred the pellet fraction than the soluble fraction, but for catL, the opposite was true, with a greater amount of catL in the soluble fraction than in the pellet.

### Cathepsin K cannibalized cathepsin L when co-incubated in the absence of substrate

**Figure 3:**
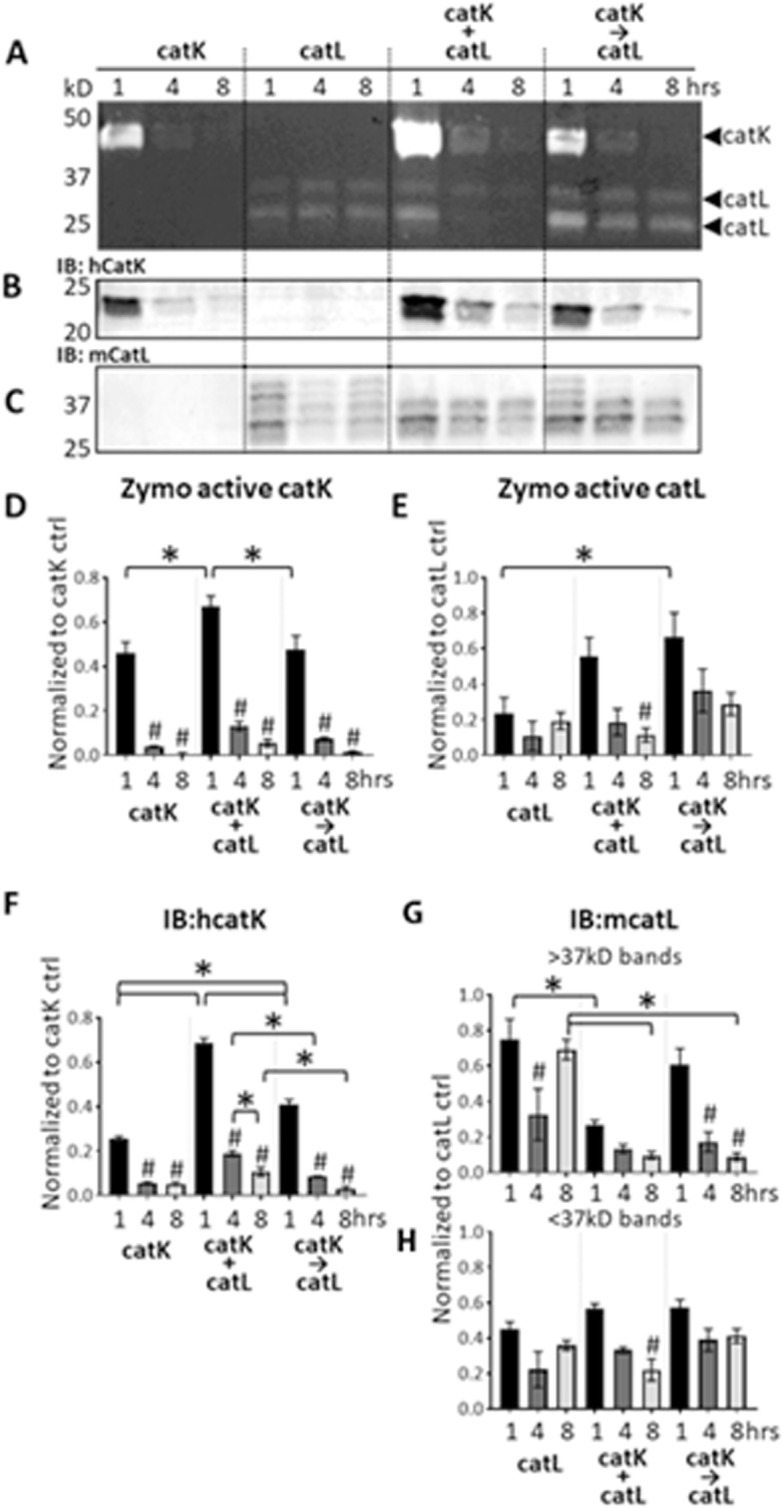
CatK cannibalized catL when co-incubated in the absence of substrate. Zymography (**A**) Immunoblot for catK (**B**) or catL (**C**) are shown. Densitometry quantified zymo active catK (**D**) catL €, or immunoblot for catK (**F**) catL >37 kDa (**G**) and catl <37 kDa (**H**). CatK and catL were incubated without substrate for 1, 4, and 8 hrs either alone, concurrently (+), or sequentially (→). Zymography showed active catK and catL remaining after incubation (**A**). CatK and catL are active at 1 hr, with 4 hr and 8 hr time points showing decreasing signal. Significantly more catK was active when concurrently co-incubated with catL (+) compared to catK alone and sequential co-incubation of catK then catL (→). However, active catL significantly decreased over time when co-incubated with catK. Significantly more catK was detected by Western blot when co-incubated with catL compared to catK alone. CatL amount significantly decreases over time when co-incubated with catK, and even when catL was added sequentially, suggesting catK cannibalism of catL. (n = 3, *p < 0.05 across conditions, ^#^indicates statistical significance from the corresponding 1 hr bar within the same condition, p < 0.05 as determined by a one-way ANOVA. Error bars represent SEM.

After finding that active catK was still present in soluble and pellet fractions after 8 hours incubation with collagen gels, it suggested that substrate increases the stability of proteases, whereas it has been reported that with soluble substrates or short peptide substrates, they were inactivated over time periods of two hours^37^. To determine if substrate was influencing cathepsin-on-cathepsin dynamics during the co-incubation conditions, catK and catL were incubated alone or together, in the absence of any substrate. When catK was incubated alone, catK autodigestion occurred as indicated by the loss of the active catK band over time in the zymogram, even in the absence of any other protease (Fig. 3A), corroborated by the loss of catK protein over time in the immunoblots (Fig. 3B). Some catL autodigestion occurred, but not to the same extent as catK (Fig. 3A,C). When catK and catL were concurrently co-incubated, in the absence of substrate, there was a significant loss of active catL signal in the zymogram by 8 hrs that was not seen when catL was incubated alone.

Next, sequential addition of catL to catK in the absence of substrate was tested to determine the outcomes on active and protein amounts of the cathepsins. CatK was incubated alone for 4 hours, then catL was added for the remaining 4 hours to match the protocols of the collagen gel experiments. In the absence of substrate, there was a significant loss of detectable active catK by zymography and total catK protein by immunoblot after 8 hrs (Fig. 3A,B), which differed from what occurred in the presence of collagen substrate when catK was still detected after 8 hours in the soluble fraction after sequential addition of catL (Fig. 2A). This suggests again that the substrate is stabilizing the active cathepsin, retaining its active form. Shorter sequential co-incubation periods of 1 and 4 hr were tested with accompanying shorter catK pre-incubation periods of 0.5 and 2 hours prior to addition of catL, respectively. A time dependent loss of catK was observed, but catL, on the other hand, under sequentially added conditions, showed no significant loss, similar to when it was incubated in the presence of collagen (Fig. 3A,C). Densitometry to quantify all bands are shown (Fig. 3D–H).

### Cathepsin L did not significantly hydrolyze tendon ECM without cathepsin K

Our central hypothesis of this study was that priming of collagen I by catK would cleave and expose additional sites susceptible to hydrolysis by catL, but the question remained of whether catL could prime for greater cleavage by catK or if this was just an effect of adding fresh protease to the system. To test this hypothesis, tendon ECM was incubated with the following catK/catL combinations: (1) primed with catK, then incubated with catL, (2) primed with catL, then incubated with catK, (3) primed with catK, then incubated with fresh catK, and (4) primed with catL, then incubated with fresh catL. Each priming/preincubation period was 4 hrs, then the subsequent addition of the secondary protease for an additional 4 hrs as was done above.

**Figure 4:**
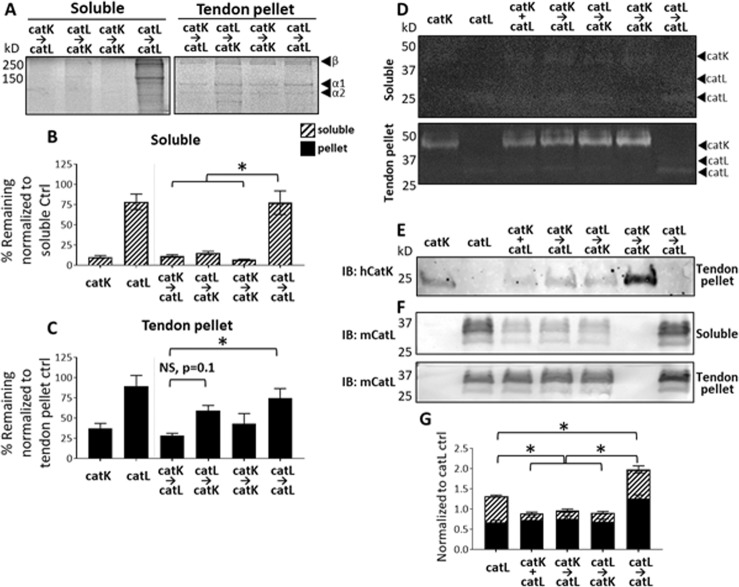
CatK as primary or secondary protease in sequential co-incubation causes higher tendon ECM degradation even during catL on catK cannibalistic interactions. Tendon ECM degradation and active catK and catL were evaluated after sequential co-incubations with either catK or catL as the primary protease. SDS-PAGE showed that catK was necessary for significant degradation of released (soluble) tendon ECM fragments (**A**,**C**) but sequential catK → catL was responsible for significant degradation of the tendon ECM pellet. Zymography shows amount of active cathepsins in the soluble fraction, and the active cathepsins that remained associated with the tendon ECM pellet. (**D**) Immunoblots for catK detected its presence in pellet fraction with less catK remaining in the presence of catL either concurrently or sequentially added. (**E**) Immunoblots for catL showed that incubation conditions with catK did not affect amounts of catL protein in the tendon ECM pellet fraction, but significantly less catL was present in the soluble fraction when catL was co-incubated with catK. (**F**,**G**) n = 3, *indicates statistical significance, p < 0.05 as determined by a one-way ANOVA. Error bars represent SEM.

Tendon ECM degradation was assessed with SDS-PAGE as done earlier. For the soluble fraction, priming with catK, and subsequent incubation with catL led to 89% of the tendon ECM being degraded leaving only 11%, and when catK was the secondary protease after priming with catL, 15% of ECM remained compared to the no enzyme control (n = 3, p < 0.05). Only 7% ECM remained when primed with catK and the secondary protease was fresh catK (Fig. 4A,B). However, priming with catL and sequential co-incubation of fresh catL left 77% of the ECM remaining, meaning the catL → catL only degraded 23% compared to no protease control (n = 3, p < 0.05). CatK → catK conditions degraded significantly more ECM than either of the catL priming conditions (n = 3, p < 0.05).

From the tendon ECM pellet fractions, 29% tendon ECM remained when primed with catK and then incubated with catL, significantly lower than the 74% remaining from tendon ECM that was primed with catL, and then subsequently incubated with fresh catL (Fig. 4A,C).

### Cathepsin K cannibalized cathepsin L when co-incubated in the presence of tendon ECM

Zymography was performed to assess amounts of active catK and catL under these new co-incubation conditions, and Western blots were used to probe for catK and catL total protein, together, to determine the extent and direction of cathepsin cannibalism. From the zymograms, there was little to no active catK and catL detected in the soluble fraction, but the amounts of active catK and catL in the pellet fractions did not differ among the incubation conditions. This indicates that cathepsins remaining active after the 8 hr incubation were associated with the tendon ECM pellet (Fig. 4D). The amount of active catK in the tendon ECM pellet did not differ among groups regardless of whether it was incubated alone, with catL, or even if it was the primary or secondary protease (n = 3).

Immunoblots from these samples detected catK protein (whether active or inactive) only in the tendon pellet fraction (Fig. 4E); soluble fraction blot not shown since there was no detectable signal. This corroborates the faint signal in the matching zymograms (Fig. 4D). No differences were measured in the total amount of catK present between any of the three conditions when catK was incubated with catL (Fig. 4E), but the amount was less than when catK was incubated alone with tendon ECM suggesting that cannibalistic loss of catK by catL was occurring. CatL protein was detectable in both soluble and tendon ECM pellet fractions. Incubation with catK did not affect amounts of catL protein in the tendon ECM pellet fraction, but there was significantly less immunodetectable catL protein in soluble fractions when catK was co-incubated with catL, compared to catL incubated alone, suggesting bidirectional cathepsin cannibalism was occurring with catK hydrolyzing catL as well (Fig. 4F,G; n = 3, p < 0.05).

### Soluble tendon ECM cleavage fragments can stabilize active cathepsins

The soluble fraction of tendon ECM was degraded significantly when incubated in the presence of catK. Their source was fragments generated by release from the insoluble ECM (collagen gels or tendon ECM pellet), with an assumption that catK and catL hydrolyze protein in the bulk pellet, releasing it from the bulk into the supernatant. Active protease in the supernatant could then bind and continue to hydrolyze the released fragments, or hydrolyze more ECM protein in the bulk pellet. These solubilized protein substrates could distract the active cathepsins from further cleavage of the bulk pellet, while also stabilizing the active cathepsins in the soluble fraction by providing them substrate protein, which was demonstrated earlier to sustain the active cathepsins, as shown in Fig. 2, but in the absence of ECM substrate protein, steady loss of active cathepsins occurs (Fig. 3).

To confirm that the solubilized tendon fragments were sufficient to stabilize the active catK and catL, extending their active time, even in the absence of bulk pellet ECM, we isolated just soluble ECM fragments from tendon ECM by incubating it in assay buffer with no protease for 8 hrs, non-enzymatically released soluble ECM fragments were released (soluble control, Fig. 1C). These solubilized tendon ECM fragments were then concurrently co-incubated with catK and catL, primed with catK then sequentially incubated with catL, or primed with catL and then subsequently incubated with catK. The reactions were stopped at different time points to assess the loss of active enzyme and substrate over time: (1) at 4 hr time point prior to addition of the secondary protease, (2) at the 6 hr point, 2 hrs of secondary protease co-incubation, and (3) at 8 hr time point, 4 hrs of secondary protease co-incubation.

**Figure 5:**
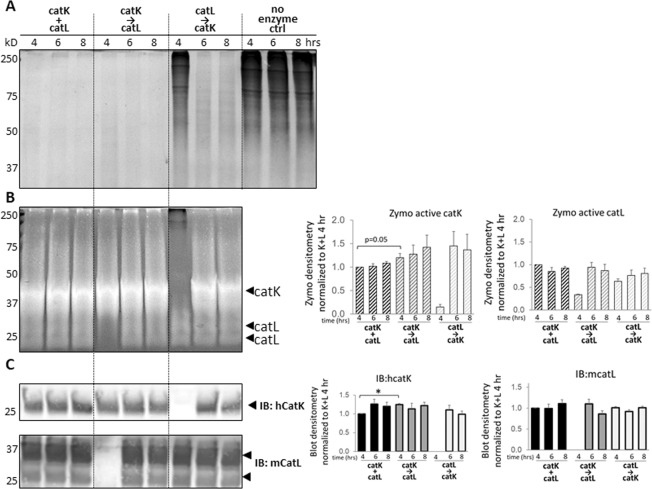
Soluble tendon ECM fragments stabilize catK and catL while being degraded, and catK is primarily responsible for tendon ECM degradation. Concurrent and sequential catK and catL were incubated with soluble tendon ECM for 4, 6, and 8 hrs. SDS-PAGE showed the soluble tendon ECM remaining after incubation time course. At all time points with either concurrent co-incubation or when primed with catK before the addition of catL, ECM fragments were below Coomassie detection. Soluble tendon ECM primed with catL was degraded only after catK was added, with fragments still visible even by 8 hrs. (**A**) Soluble tendon ECM sustained active catK and catL by zymography at all three time points. CatK was active and associated with tendon ECM fragments at multiple molecular sizes throughout the zymography gel. (**B**) Densitometry is shown on the right, and there is no significant difference in signals for these time points except for 4 and 8 hours K + L condition, (n = 3, p < 0.05). Similarly, by Western blot catK and catL were detected at similar levels at all three time points where added, with densitometry shown on the right. (**C**) Representative gels and images shown from three biological replicates.

After the periods and conditions of incubation, it was determined that soluble tendon ECM fragments were degraded below the detection limits of Coomassie staining by 4 hours when either primed with catK or when concurrently co-incubated with catK. Priming with catL did not degrade these fragments by 4 hours, but when catK was added sequentially at 4 hrs, there was degradation during the 2 and 4 hours that followed such that by the end of the incubation periods, soluble tendon ECM fragments were still visible, but depleted (Fig. 5A). Although no ECM fragments were detected in the stained polyacrylamide gel, their presence as a substrate was still sufficient to stabilize active catK and catL throughout the 6 and 8 hr periods as shown by the zymography (Fig. 5B) and by Western blots (Fig. 5C), regardless of the co- or pre-incubation condition. It is important to note that the 4 hour sequential conditions only contain one enzyme, either catL or catK, because the secondary protease is added at that time point. These time points serve as controls to what degradation is found either in the absence of catL or the absence of catK, indicated by the loss of zymography signal (Fig. 5B) and Western blot detectable signal (Fig. 5C) for each cathepsin when they are not present. Additionally, by zymography, active cathepsin bands were present around 250 kD, indicating active cathepsins were binding to larger ECM fragments as well as smaller generated fragments from their hydrolytic activity (Fig. 5B).

## Discussion

Our finding of increased cleavage of matrix substrate from the pellet fraction of collagen gels and tendon ECM when primed with catK and then incubated with catL suggest that introducing proteases to a substrate in sequence results in more efficacious matrix degradation. We have demonstrated increased hydrolysis of collagen-based matrix substrate with sequential co-incubation of catK and catL compared to concurrent co-incubation of catK and catL (Fig. 1), with evidence of protease stabilization in the presence of substrates (Fig. 2). This stabilization is partly due to the substrate-dependent cannibalistic behaviors between catK and catL that were revealed here (Fig. 3). Cathepsin cannibalism also means that catK and catL proteolyzing each other can have the net effect of reducing the amount of active proteases in the system, reducing amount of substrate degraded. Even though priming tendon ECM with catK and then incubating with catL did not hydrolyze more of the bulk substrate than the inverse of priming with catL then adding catK, priming with catK resulted in greater hydrolytic degradation of the released, soluble tendon ECM fragments (Fig. 4) and may even be necessary for this degradation (Fig. 5). In an *in vivo* tendon context, the cleavage of the pellet fraction most closely reflects the potential degradation of tendon ECM in the presence of cathepsins. However, we found that when fragments were cleaved and released into the soluble fraction, even after further degradation, these fragments were able to stabilize active cathepsins for much longer than if the proteases were without substrate (Figs 2, 3 and 5). This suggests that in the local rotator cuff tendon microenvironment, after insoluble matrix is cleaved, if not cleared away in synovial fluid, degraded fragments may be able to sustain active cathepsins for prolonged cleavage and degeneration, even if only small concentrations of cathepsins are present.

In order to understand how cathepsins cooperatively facilitate early tendon injury, the biochemical mechanisms that facilitate degeneration of tendon macromolecular structure need to be identified. Many studies have revealed how individual proteases are able to degrade extracellular matrix substrates^32,48–52^. However, it is known that cathepsins are part of a complex proteolytic network with interacting species that enhance and inhibit proteolytic potential of multiple families^37,53,54^. Given the temporal upregulation of catK and catL in the previous rat rotator cuff tendon overuse injury model^21^, the objective of this study was to investigate how concurrent and sequential co-incubations of catK and catL act in concert to degrade a type I collagen-rich substrate. The varied hydrolytic and cannibalistic interactions between catK and catL when in the presence of a collagen matrix substrate point to the complexities of the proteolytic network at play in tissue degradation. We have previously reported the propensity for cathepsin S to cannibalize catK even in the presence of a collagen substrate^37^. This is the first time we have demonstrated bi-directional cannibalism between catK and catL, even in the presence of a collagen substrate. From the observed results with the collagen substrate, more active catK was detected when co-incubated with catL than when incubated alone, suggesting that, at least initially, led to an increased amount of active catK after one hour co-incubation with catL which goes along with our hypothesis that the presence of substrate helps stabilize the active enzyme (Fig. 3B,D). Unlike catK, catL was stable over time when incubated alone. However, less catL was immunodetectable over time when co-incubated with catK (Fig. 3E), all suggesting that without substrate, catK cannibalizes catL; this cannibalistic action protects catK from its own autodigestion as evidenced by catK protein amount remaining detectable longer, since catL becomes a substrate for catK. These data point to a weighted directionality of catK on catL cannibalism when no substrate is present, however, when in the presence of collagen, catK and catL display bi-directional cannibalism (Fig. 2), suggesting that the substrate does dictate some of the proteolytic dynamics, yielding substrate-dependent responses. CatK may have a higher affinity for collagen I than catL, allowing catL the opportunity to bind catK in a cannibalistic manner, then hydrolyze it reducing the amount.

The comparison between the sequential conditions yielded more subtle differences and could be a result of the complex affinity and catalytic interactions between the cathepsins and the varying components of the tendon ECM milieu. The presence of multiple types of macromolecules in the tendon ECM besides collagen, such as elastin and proteoglycans, could have contributed to the confounding quantifications^20,52,55,56^. Studies have been published on the modifications of cathepsin activity by glycosaminoglycans, particularly in acidic conditions^32,57,58^. We and others have published that cathepsins are active by zymography while still associated with substrate^7,21,59–61^. Cathepsins can be associated or bound to the ECM by a number of other noncovalent interactions that retain its associations with the protein even in the denaturing conditions of electrophoresis. We have demonstrated this with fibrin(ogen) binding of cathepsin L^59^, and there has even been shown that cathepsin-cystatin binding is sustained under SDS-PAGE as well^62^.

This result contrasted with the zymography results from the direct incubations with the tendon ECM, where very little active catK and catL were observed in the soluble fraction (Fig. 4). This difference in cathepsin behavior may suggest that catK and catL have a higher affinity for larger tendon ECM macromolecules, but will still bind to and hydrolyze smaller, more soluble fragments. Studies have shown that certain GAGs, in addition to serving as a substrate for cathepsins, enhance cathepsin K activity^10,32,63^. Even the soluble substrate incubations served as substrate for binding and stabilizing active catK and catL with no significant difference in loss of protein or active enzyme in their presence (Fig. 5); in the absence of substrate, catK and catL used each other as substrates.

The findings of this study have many implications for understanding how cathepsins contribute to the progression of tissue destructive diseases characterized by matrix degradation. Intra-and interfamilial proteolytic crosstalk, not just the actions of a single protease, may be driving degeneration^7,21,36^. These data provide motivation to study cellular mechanisms of proteolytic upregulation. Specifically, in tendon disease, cathepsins K and L^7,21^, MMP-1, -2, and -13, and their endogenous inhibitors, are dysregulated in rat and human tendinopathy^1,16,17,19^. Further studies on the interactive effects of cathepsins and MMPs on tendon degeneration are necessary to determine an appropriate strategy for preventing disease progression. Cathepsins are very tightly regulated proteases for deliberate substrate degradation, but can display substrate promiscuity^10,24,27,64^. The stabilization of active catK by the presence of either a collagen substrate or catL, in a way that is not observed when incubated alone, suggests that even brief upregulation of secreted and active catK in a tendon injury context can result in substantial matrix degeneration. We accept that one limitation of this study is the use of homogenized tendon ECM as a test bed for degradation and active enzyme levels in co-incubation scenarios. This system does not completely recapitulate proteolytic actions on fully organized tendon, but helped to eliminate any confounding proteolytic impact from intracellular proteases present in resident tenocytes.

Another potential limitation was the use of tendon ECM from ApoE knockout mice, which have been reported to have altered baseline biomechanics of patellar and supraspinatus tendons^65,66^. Those studies were observed in much older mice, aged 10 month old, but the mice used in this study were between 2–4 months of age. There were no differences in baseline modulus observed between the ApoE deficient mice and the controls at these younger ages (14 weeks in their study)^65^, negating any confounding concerns for this study. Furthermore, the current study used homogenized tendons, not for biomechanical analyses, but as a source of relevant tissue-based collagen/ECM providing a more complicated mixture of ECM protein substrates for cathepsin binding and degradation than the collagen gels.

Although difficult to identify *in vivo*, the findings here on cannibalistic behaviors of catK and catL, particularly in the presence of a collagen substrate, suggest that cathepsin cannibalism could be a potential regulatory mechanism to restrict potent collagenases from excessive matrix hydrolysis. This suggests that if only one cathepsin is primarily facilitating matrix degradation, only that cathepsin would be the ideal target of inhibition. Alternatively, because of the interconnected behaviors of proteases within the proteolytic network, this could imply that inhibiting only one protease at one time, or even one family of proteases, may be insufficient to arrest the progression of disease. We also have shown that direct cathepsin inhibition may inhibit certain cathepsins, but result in counterintuitive upregulation of others^67^. Introduction of inhibitors specific to cathepsins and/or MMPs may have unintended consequences for tendon tissue homeostasis, and thus this impact will have to be elucidated in future experiments.
